# Supplementary material for: CSL controls telomere maintenance and genome stability in human dermal fibroblasts
Source: Nat Commun. 2019 Aug 29;10:3884. doi: 10.1038/s41467-019-11785-7 (PMC6715699; doi:10.1038/s41467-019-11785-7)
Supplement: Supplementary file 1 — Supplementary Information [file 41467_2019_11785_MOESM1_ESM.pdf]

Supplementary Information

CSL controls telomere maintenance and genome stability in human dermal fibroblasts.

Bottoni et al.

SUPPLEMENTARY FIGURE 1

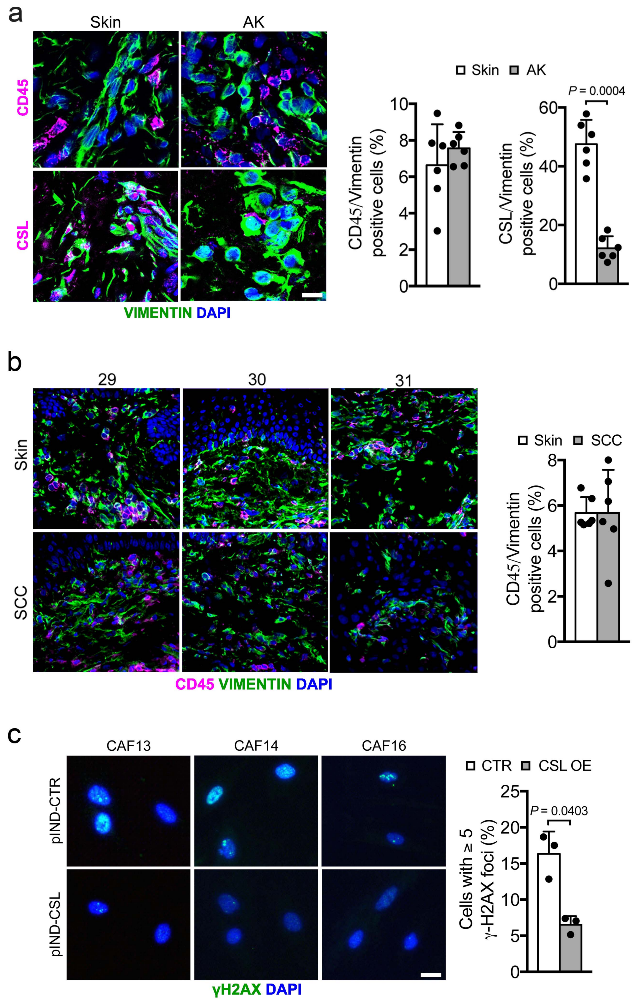

SUPPLEMENTARY FIGURE 1, related to FIGURE 2.

Analysis of CSL and leukocyte infiltration in the stroma of AK and SCC lesions; *CSL* overexpression rescues the DNA damage in CAFs.

a, CD45 / CSL (magenta) and VIMENTIN (green) immunostaining in the stroma of AKs and flanking unaffected skin. Same fields from consecutive sections as in Fig. 2a are shown. Scale bar, 10 µm. >102 VIMENTIN positive cells were counted per sample. *n*(AK/Skin)=6, ***p<0.001, two-tailed paired t-test. **b,** CD45 (magenta) and VIMENTIN (green) immunostaining in the stroma of SCCs versus matched flanking skin showing limited overlap of signals. Same fields from consecutive sections as in Fig. 2b are shown. Scale bar, 30 µm. >85 cells were counted per sample. *n*(SCC)=6, *n*(matched Skin)=6. **c,** γ-H2AX immunostaining in CAFs infected with an inducible *CSL* over-expressing lentivirus versus empty vector control and treated with Doxycycline (500 ng ml^{-1}) for 6 days. Scale bar, 10 µm. >149 cells were counted per sample. *n*(CAF strain)=3, *n*(matched HDF strain)=3, *p<0.05, two-tailed paired t-test. Bars represent mean ± SD.

SUPPLEMENTARY FIGURE 2

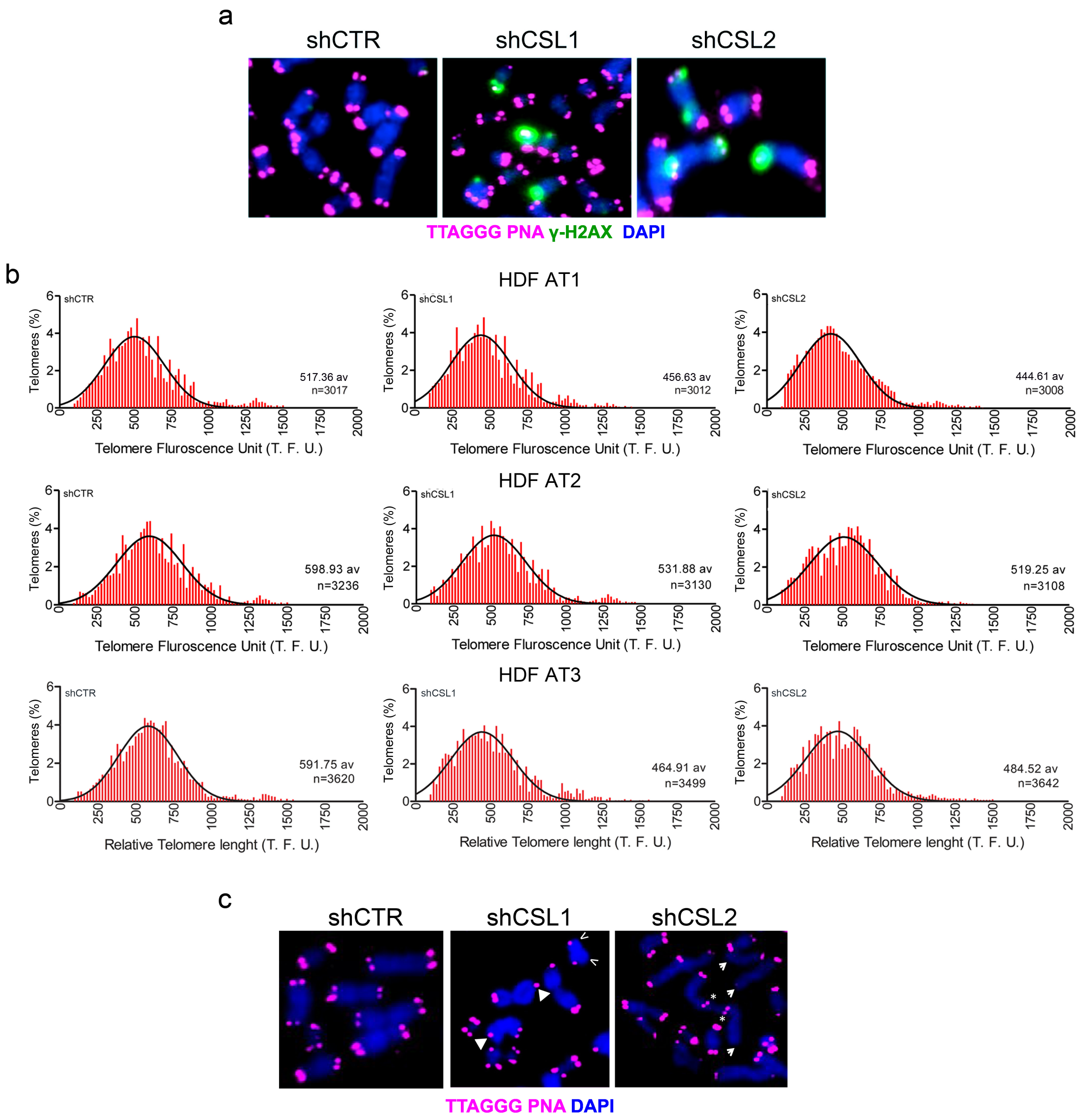

SUPPLEMENTARY FIGURE 2, related to FIGURE 3.
DNA damage induction and telomere loss in HDFs with *CSL* silencing.
a, Telomeric DNA-FISH (magenta) and γ-H2AX immunostaining (green) images of HDFs plus/minus *CSL* silencing as in Fig. 3b. **b,** Analysis of telomere length by Q-FISH in the same HDF strains plus/minus *CSL* silencing as in Fig. 3e. Histograms show the distribution of relative telomere lengths in 3 HDF strains plus/minus *CSL* silencing expressed as fluorescence intensity (AU) as in 58. A minimum of 100 TFU was set as the cut-off. Average values are shown on the right. >3000 telomeres were quantified per sample. *n*(strain)=3. **c,** Telomeric DNA Q-FISH showing chromosomal ends aberrations in HDFs plus/minus *CSL* silencing as in Fig. 3f,g. Arrowheads, triangles, arrows and stars point to OTL, SCF, TD and EJ, respectively.

SUPPLEMENTARY FIGURE 3

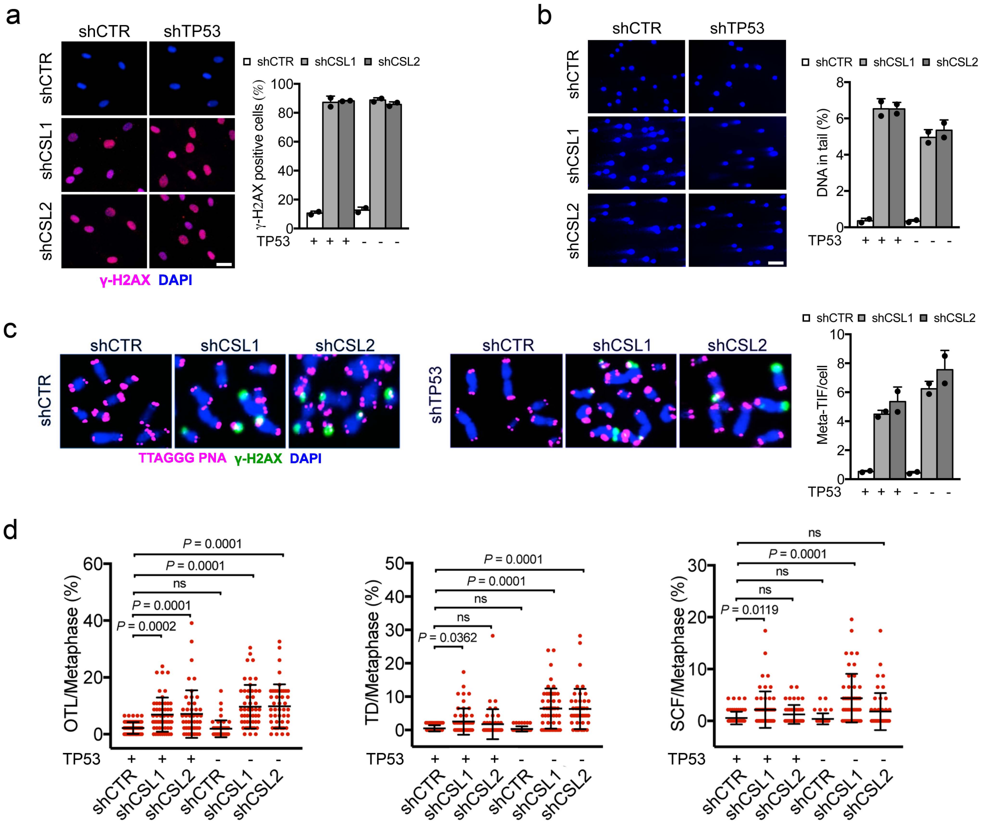

SUPPLEMENTARY FIGURE 3.

DNA damage induction and telomere loss in HDFs with *CSL* KD plus/minus *TP53* gene silencing.

a, γ-H2AX (magenta) immunostaining in HDFs plus/minus *CSL* and *TP53* silencing individually and in combination. Scale bar, 15 μm. >175 cells were counted per sample. *n*(strain)=2. **b,** Comet assays of HDFs plus/minus *CSL* and *TP53* silencing individually and in combination. Scale bar, 50 μm. >120 cells were analyzed per sample. *n*(strain)=2. **c,** Telomeric DNA Q-FISH (magenta) and γ-H2AX immunostaining (green) co-localization signals in metaphase chromosome spreads (Meta-TIF) from HDFs plus/minus *CSL* and *TP53* silencing individually and in combination. >30 spreads per sample were scored. *n*(strain)=2. **d,** Quantification of the percentage of chromosomes carrying OTLs, TDs and SCFs per metaphase in HDFs plus/minus *CSL* and *TP53* silencing individually and in combination. Mean ± SD, *n*(spread)=45, *n*(strain)=2, *p<0.05, one-way ANOVA. Bars represent mean ± SD.

SUPPLEMENTARY FIGURE 4

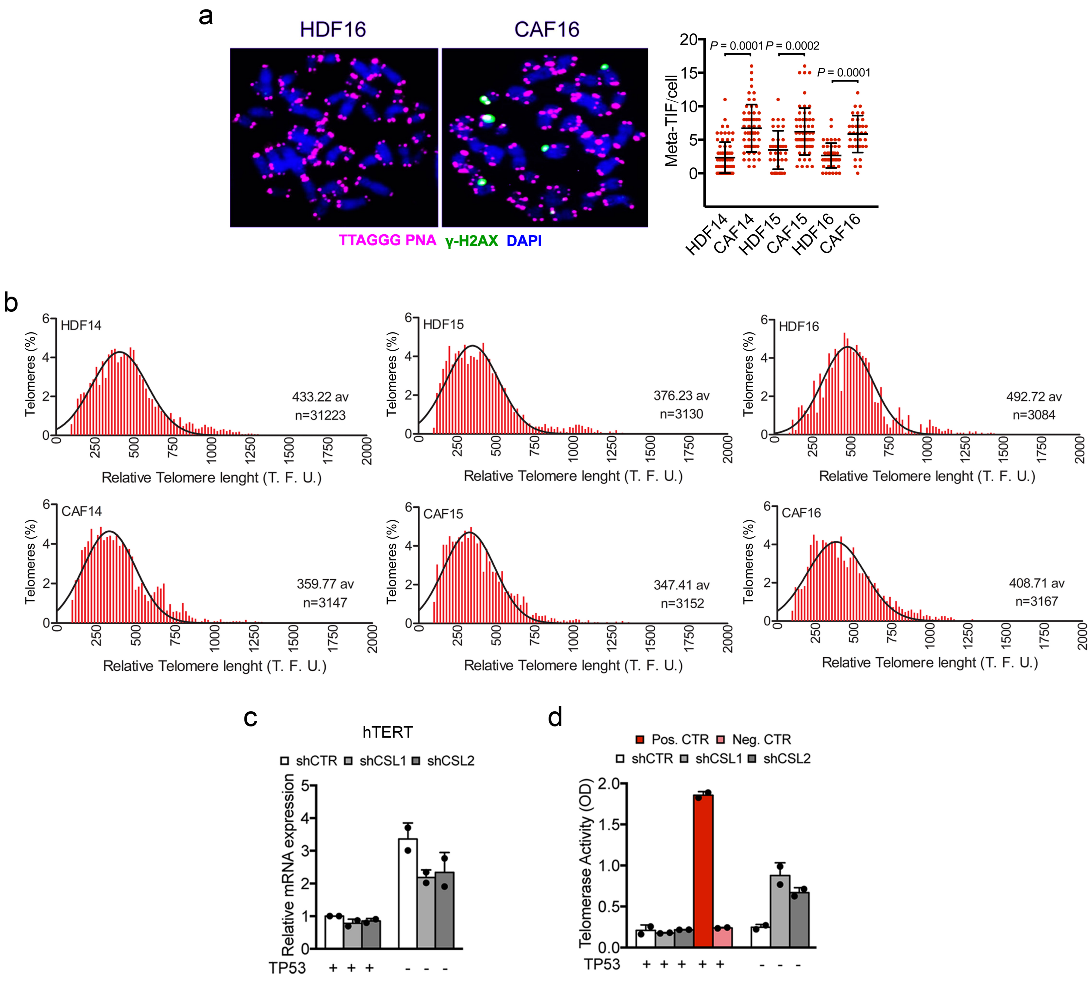

SUPPLEMENTARY FIGURE 4, related to FIGURE 4.
CAFs display telomere loss; hTERT is reactivated by concomitant *CSL* and *TP53* silencing.
a, Telomeric DNA Q-FISH (magenta) and γ-H2AX immunostaining (green) co-localization foci (Meta-TIF) in metaphase spreads of 3 CAF and matched HDF strains. *n*(spread)=50, *n*(CAF strain)=3, *n*(matched HDF strain)=3, ***p<0.001, two-tailed unpaired t-test. **b,** Analysis of telomere length by Q-FISH in the same CAF and matched HDF strains as in **a**. Histograms show the distribution of relative telomere lengths in 3 CAF and matched HDF strains expressed as fluorescence intensity (AU) as in [58]. A minimum of 100 TFU was set as the cut-off. Average values are shown on the right. >3000 telomeres were quantified per sample. *n*(CAF strain)=3, *n*(matched HDF strain)=3. **c,** RT-qPCR of *hTERT* expression, normalized to *RPLP0*, in HDFs plus/minus *CSL* and *TP53* silencing individually and in combination. *n*(strain)=2. **d,** Telomerase activity measured in HDFs plus/minus *CSL* and *TP53* silencing individually and in combination. Positive and negative controls were performed as explained in the Methods section. *n*(strain)=2. Bars represent mean ± SD.

SUPPLEMENTARY FIGURE 5

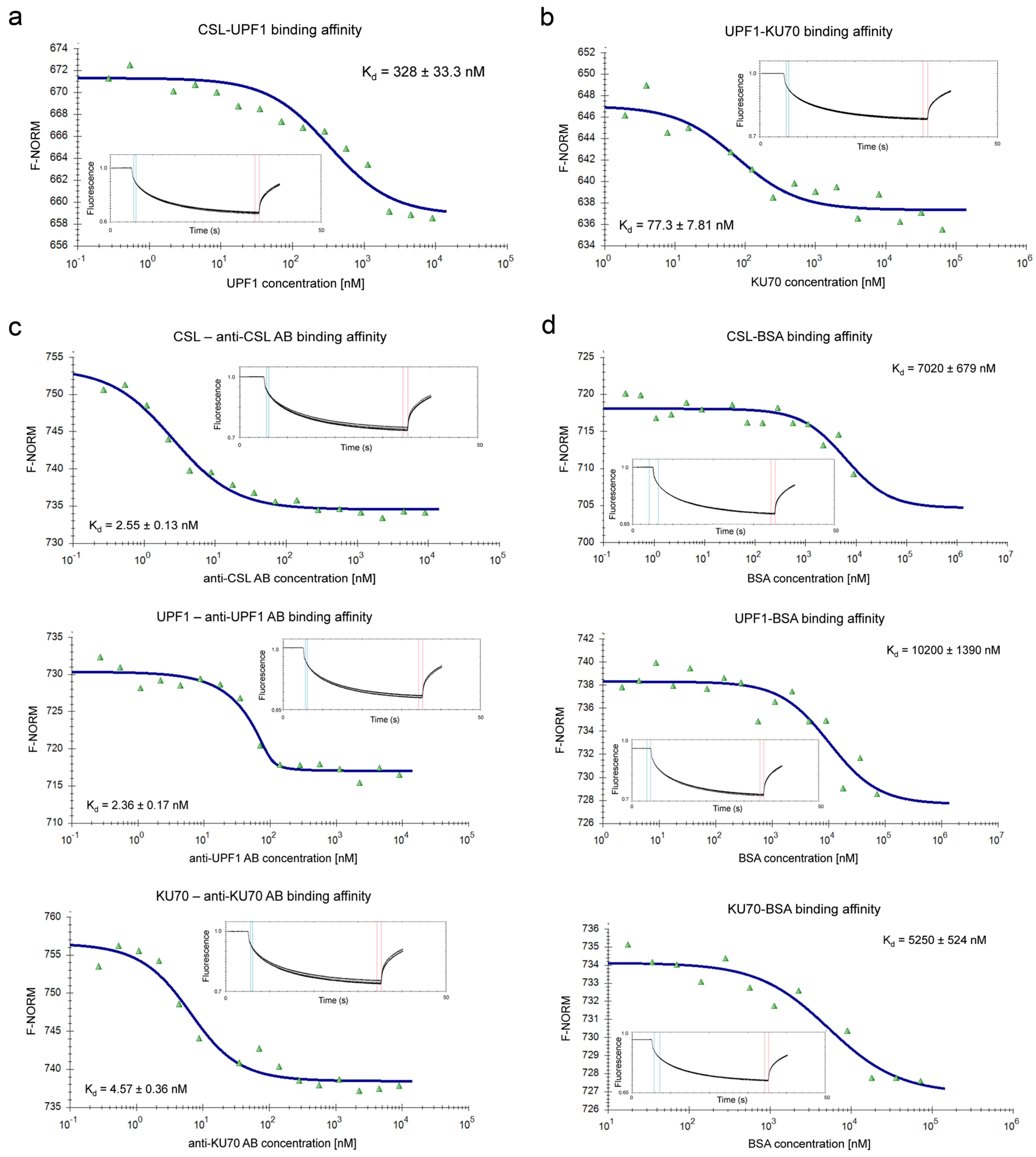

SUPPLEMENTARY FIGURE 5, related to FIGURE 5.
UPF1 direct binding to CSL and Ku70; MST specificity controls.
a-b, Binding of recombinant UPF1 to CSL **(a)** or Ku70 **(b)** proteins as measured by MST. Inset: thermophoretic movement of fluorescently-labeled CSL or UPF1. **c,** MST analysis of positive controls for high binding affinity. Each recombinant protein labeled with RED-NHS was admixed (1 μM) with 2-fold serial dilutions of the corresponding antibodies (from 9 μM to 0.27 nM). **d,** MST analysis of negative controls. Purified proteins labeled with RED-NHS were admixed with 2-fold serial dilutions of bovine serum albumin (from 72μM to 2.19 nM). Results are expressed as normalized thermophoresis-dependent fluorescence units (F-Norm) as a function of unlabeled ligand concentrations as in Fig. 5e.

SUPPLEMENTARY FIGURE 6

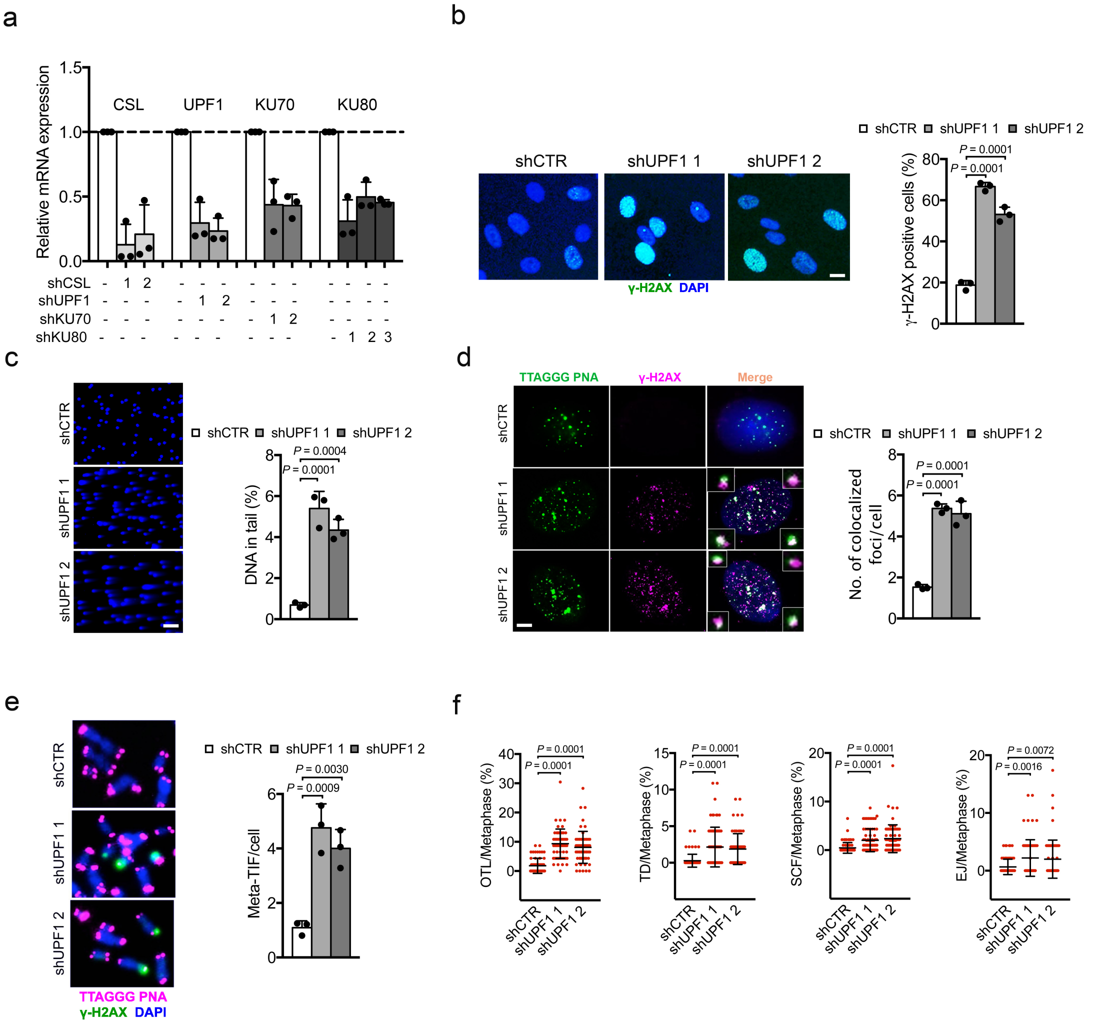

SUPPLEMENTARY FIGURE 6, related to FIGURE 5.
CSL and UPF1 play overlapping roles in telomere binding and protection.
a, RT-qPCR of *CSL/UPF1/Ku70/Ku80* mRNA expression, normalized to *RPLP0*, in HDFs plus/minus their respective genes silencing for 6 days. *n*(strain)=3. **b,** γ-H2AX immunostaining in HDFs plus/minus infection with two *UPF1* silencing lentiviruses versus empty vector control for 5 days. Scale bar, 5 μm. >124 cells were counted per sample. *n*(strain)=3, ****p=0.0001, one-way ANOVA. **c,** Comet assays of HDFs plus/minus shRNA-mediated *UPF1* silencing for 6 days. Scale bar, 50 μm. >150 cells were analyzed per sample. *n*(strain)=3, ***p<0.001, one-way ANOVA. **d,** Telomeric DNA Q-FISH (green) and γ-H2AX immunostaining (magenta) co-localization foci in HDFs plus/minus *UPF1* silencing (5 days). Scale bar, 2 μm. >50 cells per sample were scored. *n*(strain)=3, ****p=0.0001, one-way ANOVA. **e,** Telomeric DNA Q-FISH (magenta) and γ-H2AX immunostaining (green) co-localization signals in metaphase chromosome spreads (Meta-TIF) from HDFs plus/minus *UPF1* silencing as in **c**. >40 spreads per sample were scored. n(strain)=3, **p<0.01, one-way ANOVA. **f,** Quantification of the percentage of chromosomes carrying chromosomal aberrations per metaphase (OTL, TD, SCF and EJ, as defined in Fig. 3f,g) in 3 HDF strains plus/minus *UPF1* silencing for 6 days. Mean ± SD, *n*(spread)=35, *n*(strain)=3, **p<0.01, one-way ANOVA. Bars represent mean ± SD.

SUPPLEMENTARY FIGURE 7

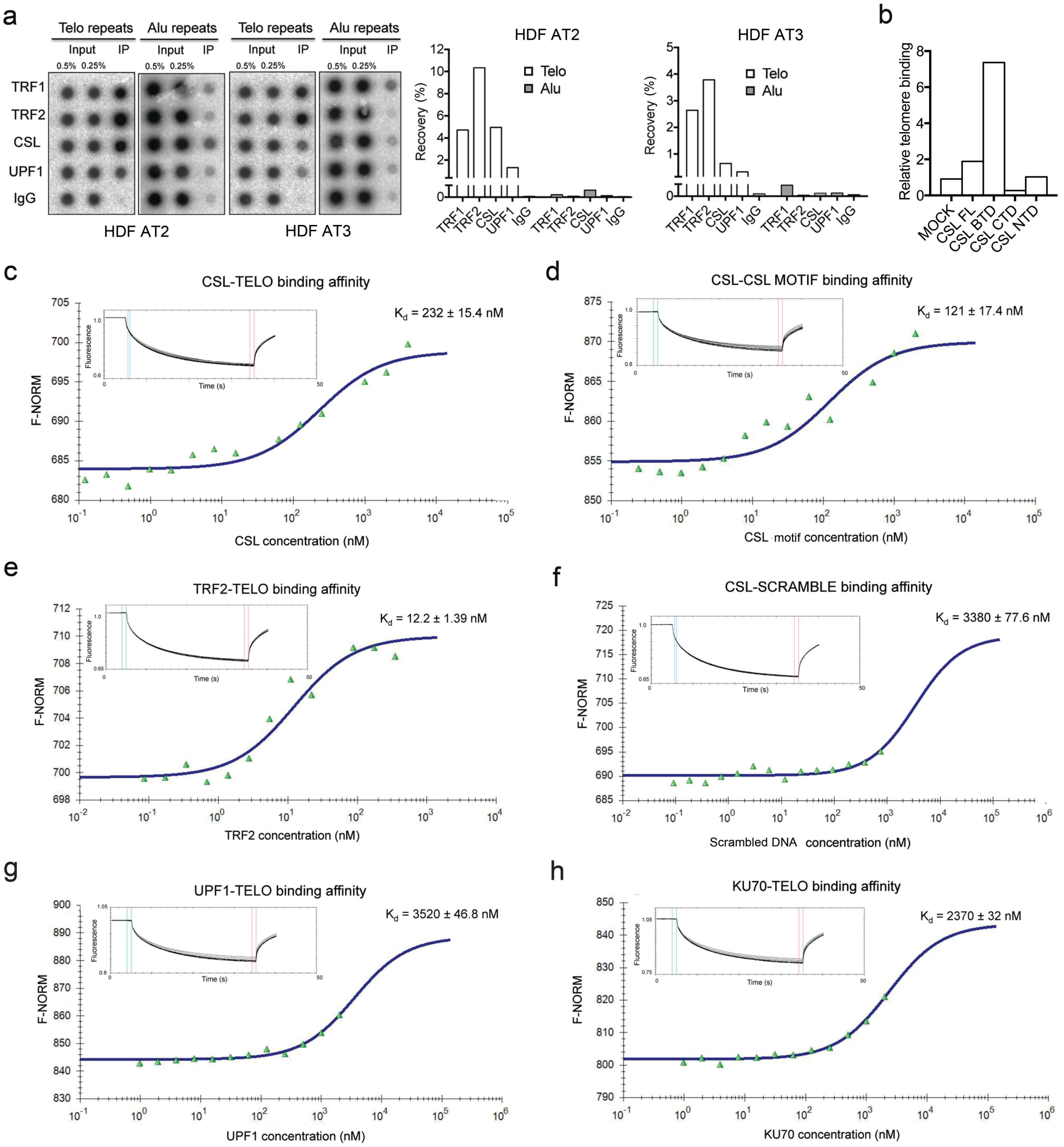

SUPPLEMENTARY FIGURE 7, related to FIGURE 6.
CSL binds to telomeres.

a, Densitometric quantification of ChIP assays with antibodies against the indicated proteins, in parallel with non-immune IgG, followed by DNA dot blot hybridization with probes detecting telomeric (Telo) or Alu repeats in HDFs. *n*(strain)=2. **b,** ChIP with antibodies against FLAG-tag followed by qPCR with telomere-specific primers in HEK293T cells expressing CSL full length (FL), CSL BTD, CSL CTD, or CSL NTD FLAG-tagged domains. Non-immune IgGs were used for normalization. **c-d,** Binding of recombinant CSL protein and telomeric sequence (TELO) or CSL motif as measured by MST. Inset: thermophoretic movement of fluorescently-labeled TELO or CSL. **e,** Binding of recombinant TRF2 protein and telomeric sequence (TELO) as measured by MST. Inset: thermophoretic movement of fluorescently-labeled TELO. MST analysis used as a positive control of Fig. 6e. **f,** Binding of recombinant CSL protein and scrambled DNA sequence as measured by MST. Inset: thermophoretic movement of fluorescently-labeled CSL. MST analysis used as a negative control of Fig. 6e. **g-h,** Binding of recombinant UPF1 **(g)** or Ku70 **(h)** proteins and telomeric sequence (TELO) as measured by MST. Inset: thermophoretic movement of fluorescently-labeled TELO.

SUPPLEMENTARY FIGURE 8

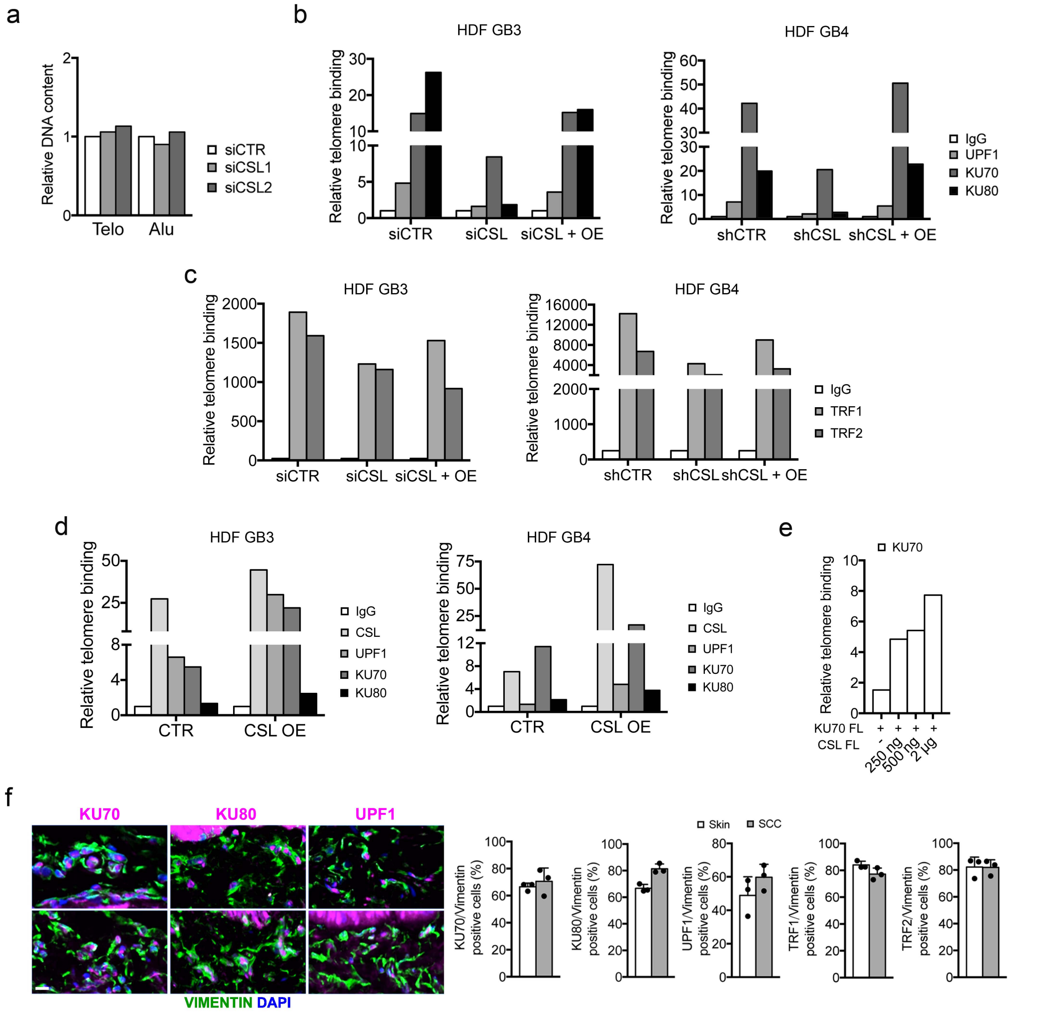

SUPPLEMENTARY FIGURE 8, related to FIGURE 7.
CSL is an essential element of a telomere binding complex.
a, qPCR analysis of telomeric and alu repeats, normalized to *ALBUMIN*, in HDFs plus/minus *CSL* silencing (3 days). **b,** Telomere binding assays by ChIP/qPCR with antibodies against the indicated proteins or non-immune IgG in HDFs plus/minus siRNA (GB3) or shRNA (GB4) mediated *CSL* silencing (3 days), and with *CSL* silencing and concomitant lenti-virally induced *CSL* overexpression (OE). *n*(strain)=2. **c,** Telomere binding assays by ChIP/qPCR analysis of the same cells as in **b** with antibodies against TRF1/TRF2. *n*(strain)=2. **d,** Telomere binding assays by ChIP/qPCR with antibodies against the indicated proteins in HDFs (GB3 and GB4) plus/minus lentivirally induced *CSL* overexpression(OE) for 3 days. *n*(strain)=2. **e,** Telomere binding assays by ChIP/qPCR with antibodies against FLAG-TAG, in parallel with non-immune IgG, in HEK293T cells expressing increasing amounts (0, 250 ng, 500 ng, 2 µg) of CSL FL FLAG-tagged together with Ku70 FL (2 µg). **f,** UPF1/Ku70/Ku80 (magenta) and VIMENTIN (green) immunostaining in the stroma of SCCs versus matched flanking skin. Same samples as in Fig. 2b are shown. Scale bar, 15 µm. >85 cells were counted per sample. *n*(SCC)=3, *n*(matched Skin)=3. Quantification of double CSL/γ-H2AX and VIMENTIN positive cells in the same samples are in Fig. 2b. Bars represent mean ± SD.

SUPPLEMENTARY FIGURE 9

a

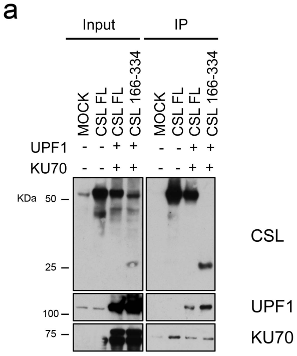

b

Mutant Position			KU70	UPF1	RAM (45)		KyoT2 (43)	RITA (42)	CIMK (44)	L3MBTL3/MBT1 (41)
Mice	Human	Interface			NCID 1	NCID 2				
R218H#(46)	R192H	BTD-DNA	-	+						
F261R	F235R	BTD	+	+	-	-	-	-	-	-
V263R	V237R	BTD	+	+	-	-	+	-	+	-
A284R	A258R	BTD	-	+	-	-	-	-	-	-
Q333R	Q307R	BTD	+	+	-	-	+	-	+	+

SUPPLEMENTARY FIGURE 9, related to FIGURE 8.
Mapping of CSL/Ku70/UPF1 interaction.
a, Co-IP analysis of HEK293T cells expressing CSL full length (FL) and CSL BTD FLAG-tagged domain with anti-FLAG magnetic beads followed by immunoblotting with antibodies against the indicated proteins. **b,** Overview of CSL-BTD domain mutants and their effects on CSL interacting partners. -, protein interaction affected by the mutation. +, protein interaction not affected by the mutation. #, CSL mutant known for its deficient DNA binding capacity. ([41-46]), Reference.

SUPPLEMENTARY FIGURE 10

Fig. 1d

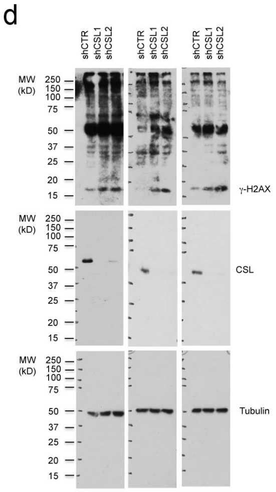

Fig. 1f

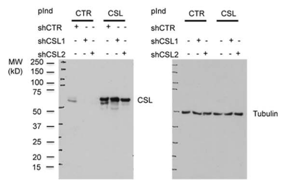

Fig. 1g

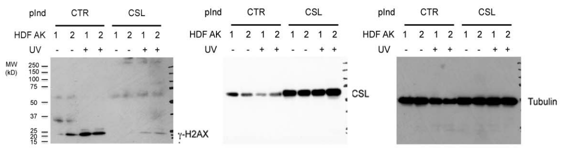

Fig. 2e

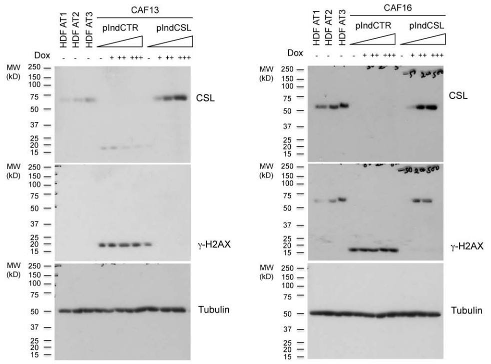

Fig. 5a

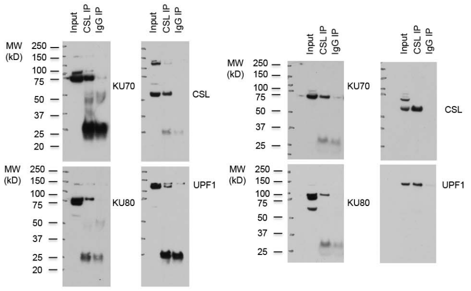

SUPPLEMENTARY FIGURE 11

Fig. 5b

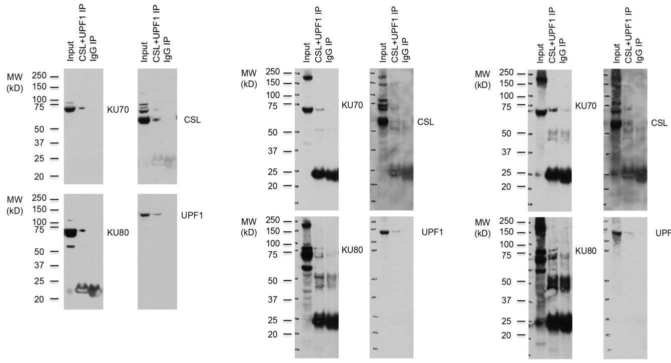

Fig. 8a

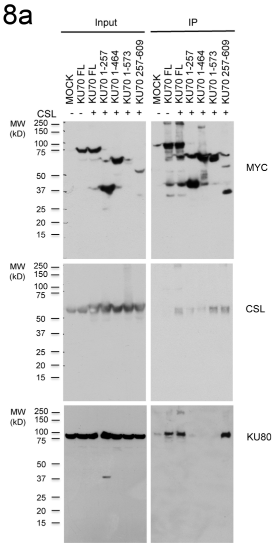

Fig. 8b

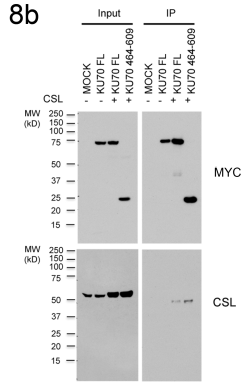

Fig. 8c

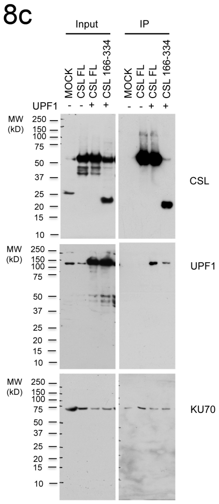

Fig. 8d

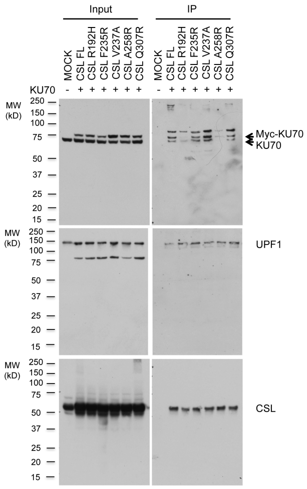

Fig. S9a

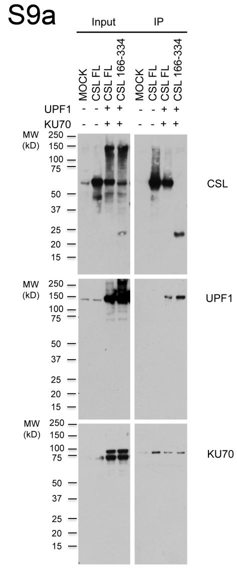

Supplementary References

41 Xu, T. *et al.* RBPJ/CBF1 interacts with L3MBTL3/MBT1 to promote repression of Notch signaling via histone demethylase KDM1A/LSD1. *EMBO J* **36**, 3232-3249, doi:10.15252/embj.201796525 (2017).

42 Tabaja, N., Yuan, Z., Oswald, F. & Kovall, R. A. Structure-function analysis of RBP-J-interacting and tubulin-associated (RITA) reveals regions critical for repression of Notch target genes. *J Biol Chem* **292**, 10549-10563, doi:10.1074/jbc.M117.791707 (2017).

43 Collins, K. J., Yuan, Z. & Kovall, R. A. Structure and function of the CSL-KyoT2 corepressor complex: a negative regulator of Notch signaling. *Structure* **22**, 70-81, doi:10.1016/j.str.2013.10.010 (2014).

44 Kim, G. S., Park, H. S. & Lee, Y. C. OPTHiS Identifies the Molecular Basis of the Direct Interaction between CSL and SMRT Corepressor. *Mol Cells* **41**, 842-852, doi:10.14348/molcells.2018.0196 (2018).

45 Chung, C. N., Hamaguchi, Y., Honjo, T. & Kawaichi, M. Site-directed mutagenesis study on DNA binding regions of the mouse homologue of Suppressor of Hairless, RBP-J kappa. *Nucleic Acids Res* **22**, 2938-2944 (1994).

46 Yuan, Z., Friedmann, D. R., VanderWielen, B. D., Collins, K. J. & Kovall, R. A. Characterization of CSL (CBF-1, Su(H), Lag-1) mutants reveals differences in signaling mediated by Notch1 and Notch2. *J Biol Chem* **287**, 34904-34916, doi:10.1074/jbc.M112.403287 (2012).

58 Feng, X. *et al.* CTC1-STN1 terminates telomerase while STN1-TEN1 enables C-strand synthesis during telomere replication in colon cancer cells. *Nat Commun* **9**, 2827, doi:10.1038/s41467-018-05154-z (2018).
