# Supplementary material for: CSL controls telomere maintenance and genome stability in human dermal fibroblasts
Source: Nat Commun. 2019 Aug 29;10:3884. doi: 10.1038/s41467-019-11785-7 (PMC6715699; doi:10.1038/s41467-019-11785-7)
Supplement: Supplementary file 3 — Description of Additional Supplementary Files [file 41467_2019_11785_MOESM3_ESM.pdf]

Description of Additional Supplementary Files

File Name: Supplementary Data 1

Description: List of genes differentially expressed upon *CSL* or *UPF1* silencing and gene ontology analysis.

File Name: Supplementary Data 2

Description: List of CSL-binding peaks on chromosome ends.

File Name: Supplementary Data 3-8

Description: List of cell strains, qPCR and RT-PCR oligonucleotides, silencer oligonucleotides, shRNA sequences and antibodies used in the study.

File name: Supplementary Movie 1

Description: Docking analysis of the CSL-Telomere DNA-Ku70/Ku80 complex shows that Ku70 C-Ku domain interacts with CSL BTD domain, while its SAP domain interacts with CSL bound to telomeric DNA. As depicted in the movie, F235R, V237R and Q307R mutants do not affect Ku interaction with neither telomeric DNA nor CSL. On the other hand, A258R mutant impairs Ku interaction with CSL, while the defective DNA binding mutant R192H dissociates Ku70/Ku80 interaction with CSL and the binding of the latter with telomeric DNA. Our modeling suggests that R192H

and A258R mutants are essential for CSL interaction with Ku70/Ku80 and telomeric DNA.
