# Supplementary material for: CSL controls telomere maintenance and genome stability in human dermal fibroblasts
Source: Nat Commun. 2019 Aug 29;10:3884. doi: 10.1038/s41467-019-11785-7 (PMC6715699; doi:10.1038/s41467-019-11785-7)
Supplement: Supplementary file 6 — Supplementary Data 3 [file 41467_2019_11785_MOESM6_ESM.pdf]

Supplementary Data 3. List of cell strains and identifiers.

**Data S3.<br>List of cell strains and identifiers**		
Cell type	Species	Name
Primary dermal fibroblasts	Human	GB1
Primary dermal fibroblasts	Human	GB2
Primary dermal fibroblasts	Human	GB3
Primary dermal fibroblasts	Human	GB4
Primary dermal fibroblasts	Human	AT1
Primary dermal fibroblasts	Human	AT2
Primary dermal fibroblasts	Human	AT3
Primary dermal fibroblasts	Human	GP1
Primary dermal fibroblasts	Human	SJ1
Primary dermal fibroblasts	Human	SG1
Primary dermal fibroblasts	Mouse	M-AT1
Primary dermal fibroblasts	Mouse	M-AT2
Primary dermal fibroblasts	Mouse	M-AT3
Primary dermal fibroblasts	Mouse	M-AT4
Primary dermal fibroblasts	Mouse	M-AT5
Primary dermal fibroblasts	Mouse	M-AT6
Cancer associated fibroblasts	Human	CAF1
Cancer associated fibroblasts	Human	CAF2
Cancer associated fibroblasts	Human	CAF11
Cancer associated fibroblasts	Human	CAF12
Cancer associated fibroblasts	Human	CAF13
Cancer associated fibroblasts	Human	CAF14
Cancer associated fibroblasts	Human	CAF15
Cancer associated fibroblasts	Human	CAF16
Matched dermal fibroblasts	Human	HDF11
Matched dermal fibroblasts	Human	HDF12
Matched dermal fibroblasts	Human	HDF13
Matched dermal fibroblasts	Human	HDF14
Matched dermal fibroblasts	Human	HDF15
Matched dermal fibroblasts	Human	HDF16
HEK 293T	Human	RRID:CVCL_0063
