# Supplementary material for: CSL controls telomere maintenance and genome stability in human dermal fibroblasts
Source: Nat Commun. 2019 Aug 29;10:3884. doi: 10.1038/s41467-019-11785-7 (PMC6715699; doi:10.1038/s41467-019-11785-7)
Supplement: Supplementary file 7 — Supplementary Data 4 [file 41467_2019_11785_MOESM7_ESM.pdf]

Supplementary Data 4. Sequence of the oligonucleotides used for qPCR experiments.

Data S4.
Sequence of the oligonucleotides used for qPCR experiments

Gene	Primer	Sequence
ALU	F	CTGGGCGACAGAACGAGATTCTAT
	R	CTCACTACTTGGTGACAGGTTCA
TELO	F	ACACTAAGGTTTGGGTTTGGGTTTGGGTTTGGGTTAGTGT
	R	TGTTAGGTATCCCTATCCCTATCCCTATCCCTATCCCTAACA
CSL mutation R192H	F	CAAAGGTGGCTCTGTTTAATCACCTACGATCCCAGACAGTTA
	R	TAACTGTCTGGGATCGTAGGTGATTAAACAGAGCCACCTTTG
CSL mutation F235R	F	GATGAATCAGAAGGAGAAGAACGCACAGTCCGAGATGGCTA
	R	TAGCCATCTCGGACTGTGCGTTCTTCTCCTTCTGATTCATC
CSL mutation V237R	F	TGAATCAGAAGGAGAAGAATTCACACGCCGAGATGGCTAC
	R	GTAGCCATCTCGGCGTGTGAATTCTTCTCCTTCTGATTCA
CSL mutation A258R	F	GTGCTCAGTTACTGGCATGCGACTCCCAAGATTGATAATT
	R	AATTATCAATCTTGGGAGTCGCATGCCAGTAACTGAGCAC
CSL mutation Q307R	F	TGCCTTTCTCAAGAAAGAATAATTCGATTTCAGGCCACTCCAT
	R	ATGGAGTGGCCTGAAATCGAATTATTCTTTCTTGAGAAAGGCA
