# Supplementary material for: CSL controls telomere maintenance and genome stability in human dermal fibroblasts
Source: Nat Commun. 2019 Aug 29;10:3884. doi: 10.1038/s41467-019-11785-7 (PMC6715699; doi:10.1038/s41467-019-11785-7)
Supplement: Supplementary file 8 — Supplementary Data 5 [file 41467_2019_11785_MOESM8_ESM.pdf]

Supplementary Data 5. Identifiers for the Silencer™ oligonucleotides (Ambion and Thermofisher) used for RNA-interference experiments.

**Data S5.**<br>**Identifiers for the Silencer™ oligonucleotides (Ambion and Thermofisher) used for RNA-interference experiments**		
Gene name	#Catalog	#ID
Control	4390846	NA
CSL siRNA #1	4392420	7252
CSL siRNA #2	4392420	7253
