# Supplementary material for: CSL controls telomere maintenance and genome stability in human dermal fibroblasts
Source: Nat Commun. 2019 Aug 29;10:3884. doi: 10.1038/s41467-019-11785-7 (PMC6715699; doi:10.1038/s41467-019-11785-7)
Supplement: Supplementary file 9 — Supplementary Data 6 [file 41467_2019_11785_MOESM9_ESM.pdf]

Supplementary Data 6. Identifiers for the shRNA sequences (Sigma) used for RNA-interference experiments.

**Data S6.**<br>**Identifiers for the shRNA sequences (Sigma) used for RNA-interference experiments**		
Human gene targeted	#ID	Sequence
CSL	#1	CCGGGCTGGAATACAAGTTGAACAACTCGAGTTGTTCAACTTGTATTCCAGCTTTTT
CSL	#2	CCGGCCCTAACGAATCAAACACAAACTCGAGTTTGTGTTTGATTCGTTAGGGTTTTT
TP53	#1	GACTCCAGTGGTAATCTACTTCAAGAGAGTAGATTACACTGGAGTCTTTTT
UPF1	#1	CCGGGCTGAGTTGAACTTCGAGGAACTCGAGTTCCTCGAAGTTCAACTCAGCTTTTT
UPF1	#2	CCGGCCAACCCGATAAACCGATGTTCTCGAGAACATCGGTTTATCGGGTTGGTTTTT
KU70	#1	CCGGGAAGAGTCTACCCGACATAAGCTCGAGCTTATGTCGGGTAGACTCTTCTTTTTG
KU70	#2	CCGGCACATACAGAAGTGACAGCTTCTCGAGAAGCTGTCACTTCTGTATGTGTTTTTG
KU80	#1	CCGGAATCTAAGAGAGCTGCCATCGCTCGAGCGATGGCAGCTCTCTTAGATTTTTTTG
KU80	#2	CCGGCGCTTTAACAACTTCCTGAAACTCGAGTTTCAGGAAGTTGTTAAAGCGTTTTTG
