# Supplementary material for: CSL controls telomere maintenance and genome stability in human dermal fibroblasts
Source: Nat Commun. 2019 Aug 29;10:3884. doi: 10.1038/s41467-019-11785-7 (PMC6715699; doi:10.1038/s41467-019-11785-7)
Supplement: Supplementary file 10 — Supplementary Data 7 [file 41467_2019_11785_MOESM10_ESM.pdf]

Supplementary Data 7. Sequence of the oligonucleotides used for qRT-PCR experiments.

**Data S7. Sequence of the oligonucleotides used for qRT-PCR experiments**		
Gene	Primer	Sequence
RPLP0	F	GCAATGTTGCCAGTGTCTGT
	R	GCCTTGACCTTTTCAGCAAG
hTERT	F	GCAGGAGCTGACGTGGAAG
	R	ACTCATCAGCCAGTGCAGGA
IL6	F	GGGCTCTTCGGCAAATGTAGCAT
	R	TGCTATCACCTCCCCTGTGTGGA
ACTA2	F	AGCGCAAATACTCTGTCTGG
	R	AGGCATAATTCCACAGGACA
POSTN	F	CAAAACTGAAGGACCCACAC
	R	TATTTCCACAGGCACTCCAT
FAP	F	AAACCCCTCAGACAGTTTGC
	R	GCTTGAACTTCTGAGTCCTC
CSL	F	ACCGCATTATTGGATGCAGA
	R	AGGAAGCGCCATCATTTATCA
UPF1	F	AGCGCCTTCCCATCCAACAT
	R	GGCCCTGGGTCACGTAGAAG
KU70	F	GGGTTCACTTTGAGGAATCCA
	R	GGCTTGAGAGCCTTCTGGAC
KU80	F	TTCATGGGAAATCAAGTTCTAAAGG
	R	ACGCCGACTTGAGGATTAGC
