# Supplementary material for: CSL controls telomere maintenance and genome stability in human dermal fibroblasts
Source: Nat Commun. 2019 Aug 29;10:3884. doi: 10.1038/s41467-019-11785-7 (PMC6715699; doi:10.1038/s41467-019-11785-7)
Supplement: Supplementary file 11 — Supplementary Data 8 [file 41467_2019_11785_MOESM11_ESM.pdf]

Supplementary Data 8. List of antibodies used.

Data S8.<br>List of antibodies used				
Target	Species	Company and catalog #	Assay and dilution used	RRID
γ -H2AX	Rabbit	Cell Signaling #2577	IF (1:100), WB (1:1000)	RRID:AB_2118010
γ-Tubulin	Mouse	Sigma #GTU-88	WB (1:2000)	RRID:AB_477584
Vimentin	Goat	R&D #AF2105	IF (1:200)	RRID:AB_355153
Vimentin	Mouse	Abcam #20346	IF (1:200)	RRID:AB_445527
CSL	Rabbit	Cell Signaling #5313	WB (1:1000), IP, ChIP	RRID:AB_2665555
CD45	Mouse	BioLegend #304001	IF (1:200)	RRID:AB_314389
CSL	Mouse	Santa Cruz #271128	PLA (1:50), IF (1:50)	RRID:AB_10610612
UPF1	Rabbit	Abcam #109363	PLA (1:50), WB (1:1000), IP, ChIP	RRID:AB_10861979
UPF1	Rabbit	Sigma #HPA019587	IF (1:100)	RRID:AB_1856174
KU70	Rabbit	GeneTex #101820	PLA (1:50), WB (1:1000), ChIP	RRID:AB_10731639
KU80	Rabbit	GeneTex #109935	PLA (1:50), WB (1:1000), ChIP	RRID:AB_1952614
TRF1	Rabbit	Chawla et al, EMBO J. 2011	ChIP	
TRF2	Rabbit	Chawla et al, EMBO J. 2011	ChIP	
UPF1	Rabbit	Chawla et al, EMBO J. 2011	ChIP	
TRF1	Rabbit	GeneTex #32935	PLA (1:50)	
TRF2	Mouse	Abcam #13579	PLA (1:50)	RRID:AB_300474
PDGFRα-FITC	Mouse	Santa Cruz #21789	IF (1:50)	RRID:AB_626904
Flag M2	Mouse	Sigma # F1804	ChIP	RRID:AB_262044
Myc-tag	Rabbit	Cell Signaling #2278	ChIP, WB (1:2000)	RRID:AB_490778
