# Supplementary material for: CSL controls telomere maintenance and genome stability in human dermal fibroblasts
Source: Nat Commun. 2019 Aug 29;10:3884. doi: 10.1038/s41467-019-11785-7 (PMC6715699; doi:10.1038/s41467-019-11785-7)
Supplement: Supplementary file 13 — Reporting Summary [file 41467_2019_11785_MOESM13_ESM.pdf]

nature research

Corresponding author(s): Gian Paolo Dotto

Last updated by author(s): Jul 19, 2019

Reporting Summary

Nature Research wishes to improve the reproducibility of the work that we publish. This form provides structure for consistency and transparency in reporting. For further information on Nature Research policies, see Authors & Referees and the Editorial Policy Checklist.

Statistics

For all statistical analyses, confirm that the following items are present in the figure legend, table legend, main text, or Methods section.

n/a	Confirmed	
☐	☒	The exact sample size (*n*) for each experimental group/condition, given as a discrete number and unit of measurement
☐	☒	A statement on whether measurements were taken from distinct samples or whether the same sample was measured repeatedly
☐	☒	The statistical test(s) used AND whether they are one- or two-sided<br>*Only common tests should be described solely by name; describe more complex techniques in the Methods section.*
☐	☒	A description of all covariates tested
☐	☒	A description of any assumptions or corrections, such as tests of normality and adjustment for multiple comparisons
☐	☒	A full description of the statistical parameters including central tendency (e.g. means) or other basic estimates (e.g. regression coefficient) AND variation (e.g. standard deviation) or associated estimates of uncertainty (e.g. confidence intervals)
☐	☒	For null hypothesis testing, the test statistic (e.g. *F*, *t*, *r*) with confidence intervals, effect sizes, degrees of freedom and *P* value noted<br>*Give P values as exact values whenever suitable.*
☒	☐	For Bayesian analysis, information on the choice of priors and Markov chain Monte Carlo settings
☒	☐	For hierarchical and complex designs, identification of the appropriate level for tests and full reporting of outcomes
☒	☐	Estimates of effect sizes (e.g. Cohen's *d*, Pearson's *r*), indicating how they were calculated

Our web collection on statistics for biologists contains articles on many of the points above.

Software and code

Policy information about availability of computer code

Data collection	N/A
Data analysis	PRISM, ImageJ, Comet Score 1.6.1.13, Bowtie2 Version 2.3.0, MACS2, HOMER, IGV, NanoTemper analysis software, Swiss-Model Expasy, HDOCK, PyMOL 2.3

For manuscripts utilizing custom algorithms or software that are central to the research but not yet described in published literature, software must be made available to editors/reviewers. We strongly encourage code deposition in a community repository (e.g. GitHub). See the Nature Research guidelines for submitting code & software for further information.

Data

Policy information about availability of data

All manuscripts must include a data availability statement. This statement should provide the following information, where applicable:
- Accession codes, unique identifiers, or web links for publicly available datasets
- A list of figures that have associated raw data
- A description of any restrictions on data availability

GSE59942 (Fig.6a), GSE113542 (Fig. 5g)

Field-specific reporting

Please select the one below that is the best fit for your research. If you are not sure, read the appropriate sections before making your selection.

☒ Life sciences ☐ Behavioural & social sciences ☐ Ecological, evolutionary & environmental sciences

Life sciences study design

All studies must disclose on these points even when the disclosure is negative.

Sample size	No statistical method was used to predetermine sample size. The samples sizes were selected based on previous studies with similar methodologies.
Data exclusions	No inclusion / exclusion criteria were adopted.
Replication	All attempts at replication were successful.
Randomization	No randomization was adopted.
Blinding	No blinding was adopted.

Reporting for specific materials, systems and methods

We require information from authors about some types of materials, experimental systems and methods used in many studies. Here, indicate whether each material, system or method listed is relevant to your study. If you are not sure if a list item applies to your research, read the appropriate section before selecting a response.

Materials & experimental systems

n/a	Involved in the study	
☐	☒	Antibodies
☐	☒	Eukaryotic cell lines
☒	☐	Palaeontology
☐	☒	Animals and other organisms
☒	☐	Human research participants
☒	☐	Clinical data

Methods

n/a	Involved in the study	
☐	☒	ChIP-seq
☒	☐	Flow cytometry
☒	☐	MRI-based neuroimaging

Antibodies

Antibodies used

γ -H2AX Rabbit Cell Signaling #2577 IF (1:100), WB (1:1000) RRID:AB_2118010
γ-Tubulin Mouse Sigma #GTU-88 WB (1:2000) RRID:AB_477584
Vimentin Goat R&D #AF2105 IF (1:200) RRID:AB_355153
Vimentin Mouse Abcam #20346 IF (1:200) RRID:AB_445527
CSL Rabbit Cell Signaling #5313 WB (1:1000), IP, ChIP RRID:AB_2665555
CD45 Mouse BioLegend #304001 IF (1:200) RRID:AB_314389
CSL Mouse Santa Cruz #271128 PLA (1:50), IF (1:50) RRID:AB_10610612
UPF1 Rabbit Abcam #109363 PLA (1:50), WB (1:1000), IP, ChIP RRID:AB_10861979
UPF1 Rabbit Sigma #HPA019587 IF (1:100) RRID:AB_1856174
KU70 Rabbit GeneTex #101820 PLA (1:50), WB (1:1000), ChIP RRID:AB_10731639
KU80 Rabbit GeneTex #109935 PLA (1:50), WB (1:1000), ChIP RRID:AB_1952614
TRF1 Rabbit Chawla et al, EMBO J. 2011 ChIP
TRF2 Rabbit Chawla et al, EMBO J. 2011 ChIP
UPF1 Rabbit Chawla et al, EMBO J. 2011 ChIP
TRF1 Rabbit GeneTex #32935 PLA (1:50)
TRF2 Mouse Abcam #13579 PLA (1:50) RRID:AB_300474
PDGFRα-FITC Mouse Santa Cruz #21789 IF (1:50) RRID:AB_626904
Flag M2 Mouse Sigma # F1804 ChIP RRID:AB_262044
Myc-tag Rabbit Cell Signaling #2278 ChIP, WB (1:2000) RRID:AB_490778

Validation

Every Cell Signalling antibody underwent an application-specific validation by the company. CSL Mouse Santa Cruz #271128, UPF1 Rabbit Abcam #109363, UPF1 Rabbit Sigma #HPA019587, KU70 Rabbit GeneTex #101820, KU80 Rabbit GeneTex #109935 were tested by shRNA silencing. All the other antibodies were previously tested and published by the authors or other groups.

Eukaryotic cell lines

Policy information about cell lines

Cell line source(s)

HDFs were prepared from discarded foreskin or abdominoplasty skin samples at the Department of Dermatology, Massachusetts General Hospital (Boston, Massachusetts, USA) with institutional approval (2000P002418), or were previously

	obtained. Pairs of CAFs and matched HDFs from discarded skin SCC and flanking unaffected areas from the same (anonymized) patients were given specific identifiers.
Authentication	HDF and CAF strains were tested in IF to be Vimentin-positive and Keratin-negative.
Mycoplasma contamination	Mycoplasma tested
Commonly misidentified lines (See ICLAC register)	N/A

Animals and other organisms

Policy information about studies involving animals; ARRIVE guidelines recommended for reporting animal research

Laboratory animals	Characterization of mice with mesenchymal Csl/Rbp-jκ deletion and mouse dermal fibroblasts (MDFs) isolation were previously reported
Wild animals	N/A
Field-collected samples	N/A
Ethics oversight	All mouse work was performed according to the Swiss guidelines and regulations for the care and use of laboratory animals with approved protocol from the Canton de Vaud veterinary office.

Note that full information on the approval of the study protocol must also be provided in the manuscript.

ChIP-seq

Data deposition

☒ Confirm that both raw and final processed data have been deposited in a public database such as GEO.

☒ Confirm that you have deposited or provided access to graph files (e.g. BED files) for the called peaks.

Data access links *May remain private before publication.*	GSE59942
Files in database submission	CSL ChIPSeq_CellSignalling Ab, CSL ChIPSeq_HomeMade Ab, Input DNA
Genome browser session (e.g. UCSC)	IGV

Methodology

Replicates	One strain of HDFs was immunoprecipitated with two antibodies against CSL
Sequencing depth	A total of 10 ng DNA were used for library preparation using NEBNext® ChIP-Seq Library Prep Reagent Set for Illumina, as recommended by the manufacturer. The samples were sequenced using Illumina HiSeq 2000 (Homo sapiens) with 100-300 bp read length and single-end sequenced.
Antibodies	5 µg of homemade and 10 µl of commercially available (Rabbit Cell Signaling #5313) antibodies were used for CSL immunoprecipitation
Peak calling parameters	Burrows-Wheeler Aligner [http://bio-bwa.sourceforge.net/] was used for fastq files alignments and, for peak detection, MACS software [http://liulab.dfci.harvard.edu/MACS/] with default parameters.
Data quality	The number of ChIP-seq peaks are approximately 25K after filter ($p<0.0001$).
Software	Burrows-Wheeler Aligner [http://bio-bwa.sourceforge.net/] was used for fastq files alignments and, for peak detection, MACS software [http://liulab.dfci.harvard.edu/MACS/] with default parameters. The Integrative Genomics Viewer (IGV) [http://www.broadinstitute.org/igv] was used for graphic illustration of ChIP-Seq peaks, and ENCODE data (http://genome.ucsc.edu/ENCODE/) for information on chromatin organization. ChIP-Seq data are deposited in the public repository (GSE59942).
